# Supplementary material for: The Structural and Electrochemical Properties of CuCoO2 Crystalline Nanopowders and Thin Films: Conductivity Experimental Analysis and Insights from Density Functional Theory Calculations
Source: Nanomaterials (Basel). 2023 Aug 11;13(16):2312. doi: 10.3390/nano13162312 (PMC10459735; doi:10.3390/nano13162312)
Supplement: Supplementary file 1 [file nanomaterials-13-02312-s001.zip › nanomaterials-2536819-supplementary.pdf]

Supplementary Information

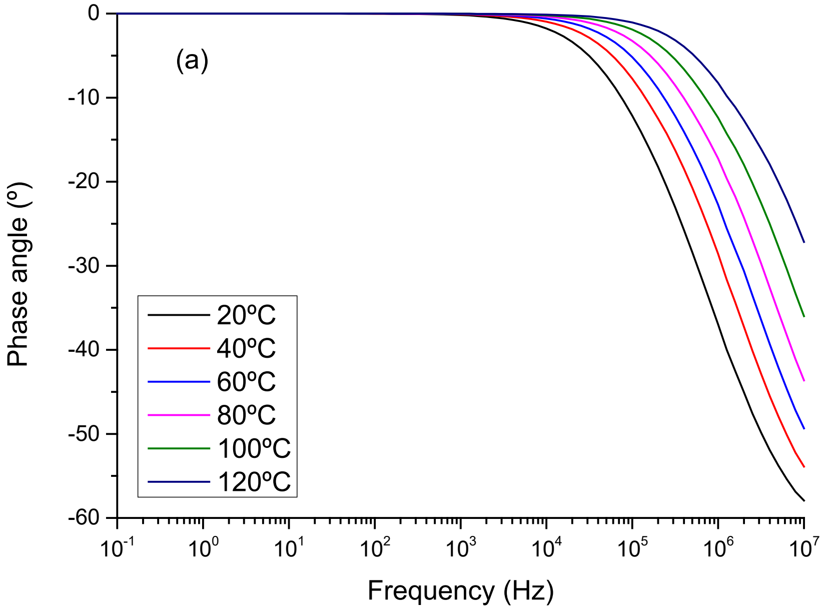

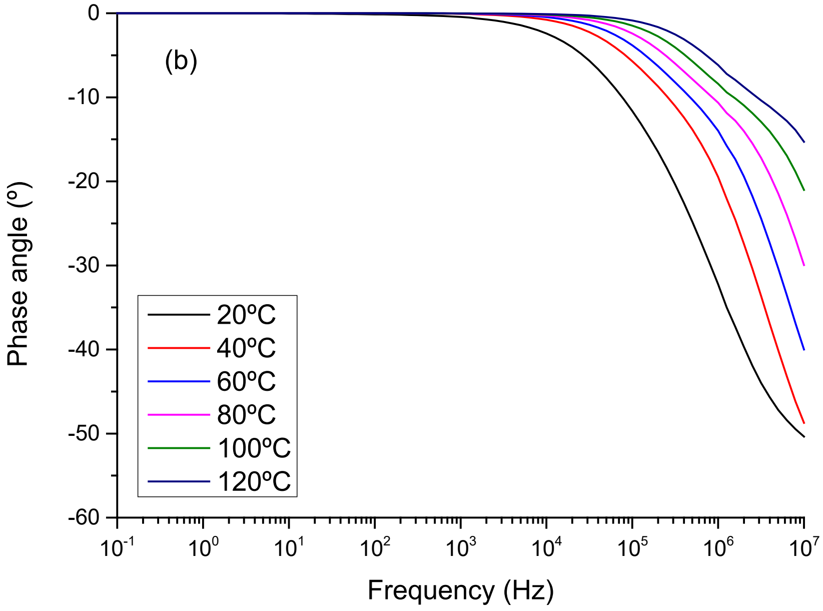

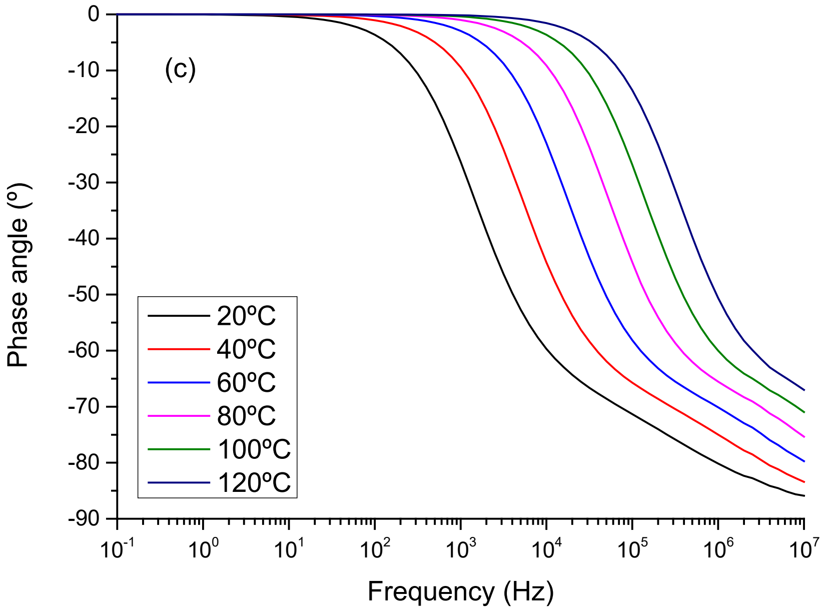

Figure S1. Phase angle (ϕ) corresponding to the Bode diagrams for (a) $CuCoO_2$_H, (b) $CuCoO_2$_SG, and (c) $CuCoO_2$_SSR delafossite materials at temperatures ranging from −20 °C to 120 °C, with increments of 20 °C, as shown.
